# Supplementary material for: Efficient breeding of industrial brewing yeast strains using CRISPR/Cas9-aided mating-type switching
Source: Appl Microbiol Biotechnol. 2021 Oct 13;105(21-22):8359–76. doi: 10.1007/s00253-021-11626-y (PMC8557189; doi:10.1007/s00253-021-11626-y)
Supplement: Supplementary file 1 — Supplementary file1 (PDF 1135 KB) [file 253_2021_11626_MOESM1_ESM.pdf]

Supplementary Figures

Journal: Applied Microbiology and Biotechnology

Efficient breeding of industrial brewing yeast strains using CRISPR/Cas9-aided mating-type switching

Kristoffer Krogerus[1*], Eugene Fletcher[2], Nils Rettberg[3], Brian Gibson[4], Richard Preiss[2]

[1] VTT Technical Research Centre of Finland, Tietotie 2, P.O. Box 1000, FI-02044 VTT, Espoo, Finland

[2] Escarpment Laboratories, Guelph, ON, Canada

[3] Research Institute for Beer and Beverage Analysis, Versuchs- und Lehranstalt für Brauerei in Berlin (VLB) e.V., Seestr. 13, 13353 Berlin, Germany

[4] Chair of Brewing and Beverage Technology, Technische Universität Berlin, Berlin, Germany

* Address correspondence to Kristoffer Krogerus, kristoffer.krogerus@vtt.fi

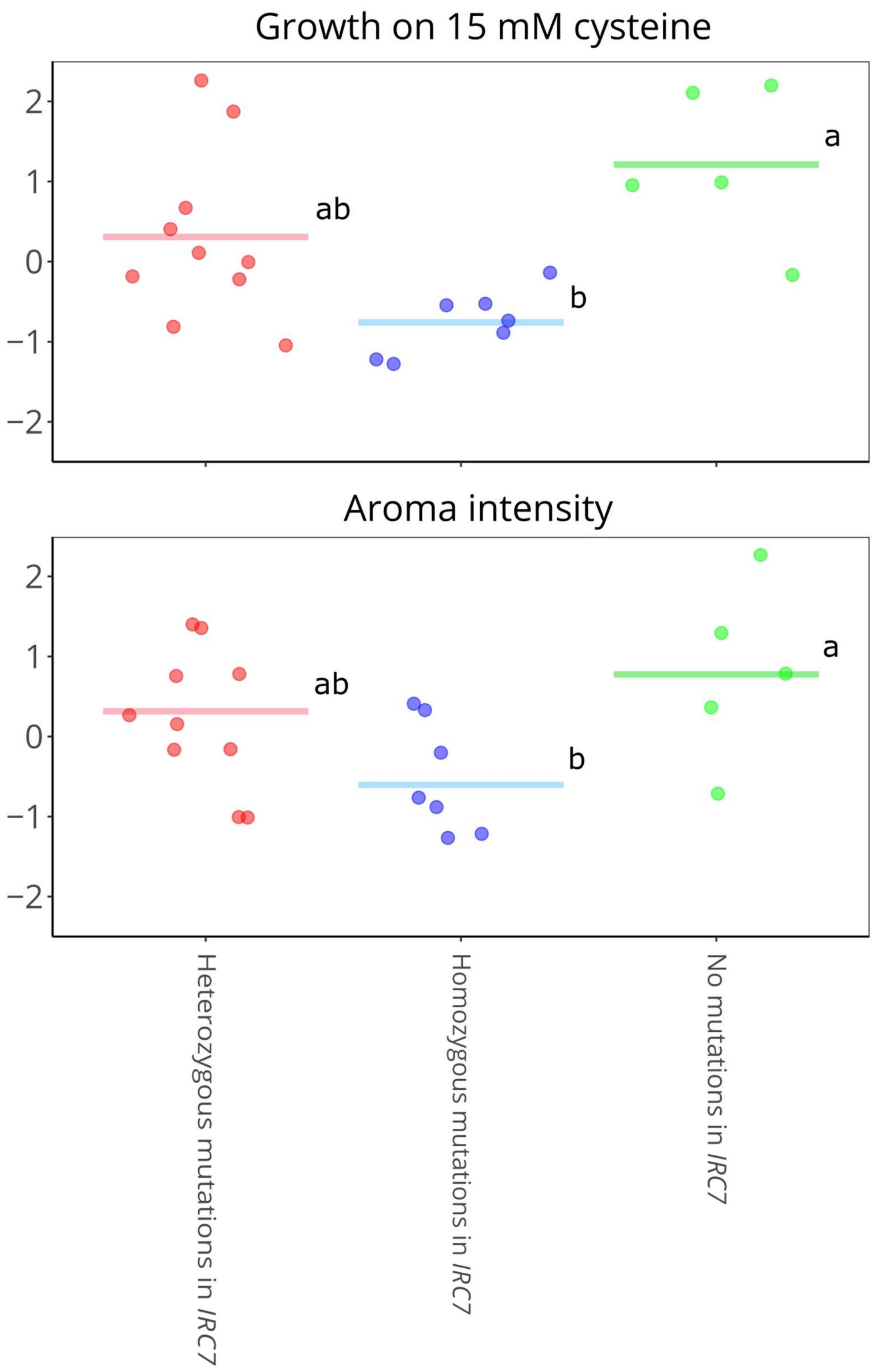

Supplementary Figure S1 - Comparison of *S. cerevisiae* strains homozygous for the *IRC7* long allele. Growth on 15 mM cysteine and aroma intensity in wort fermentations supplemented with Cys-4MMP was compared between strains harbouring either heterozygous, homozygous or no inactivating SNPs in *IRC7.* Groups with different letters differ significantly ($p < 0.05$).

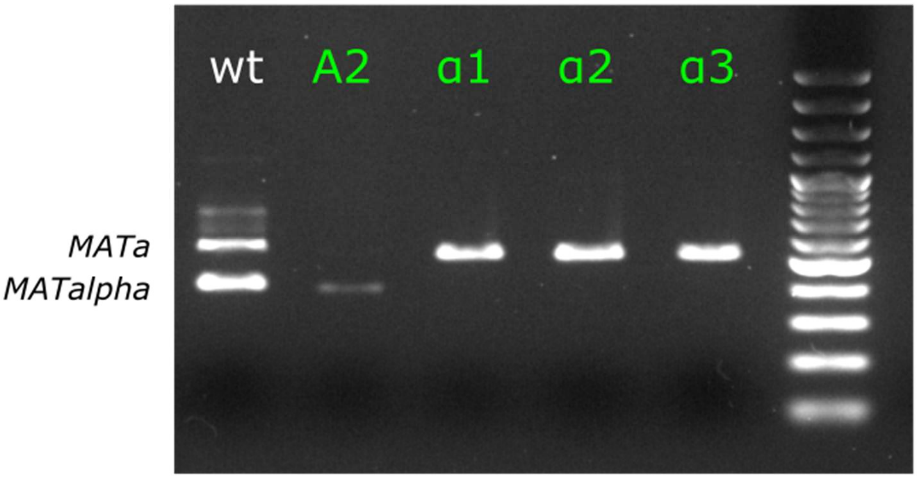

Supplementary Figure S2 - Successful mating-type change as determined by PCR. The wild-type (wt) strain is heterozygous for the mating type locus. Transformant A2 has been transformed with a Cas9 plasmid targeting *MAT***a**, and produces only a band for *MAT*α. Transformants α1-3 have been transformed with a Cas9 plasmid targeting *MAT*α, and produces only a band for *MAT***a**. Primers used: MAT-R, MATa-F and MATα-F.

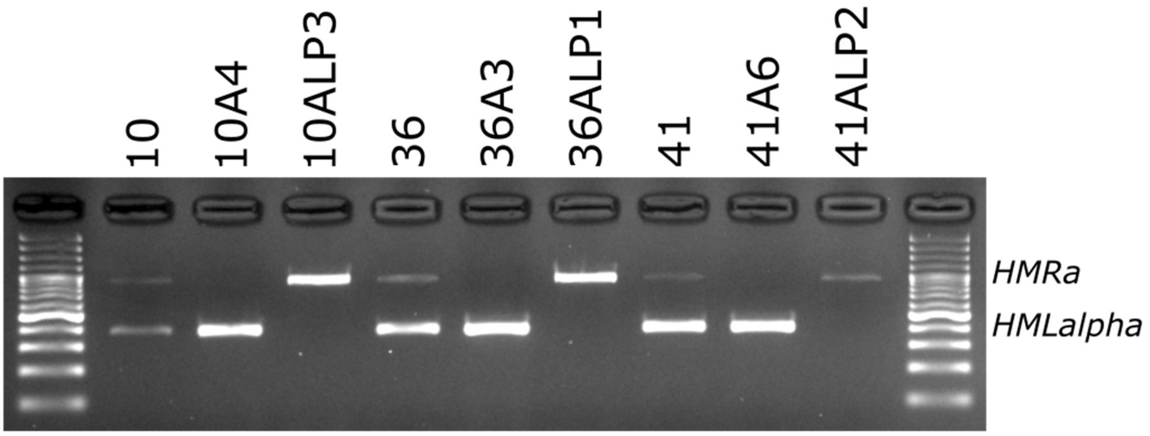

Supplementary Figure S3 - *HMR***a** and *HML*α are simultaneously deleted in the transformed strains. Wild-type strains 10, 36 and 41 produce bands for both *HMR***a** and *HML*α. A transformants (Cas9 plasmid targeting *MAT***a**) produce bands only for *HML*α. Alpha transformants (Cas9 plasmid targeting *MAT*α) produce bands only for *HMR***a**.

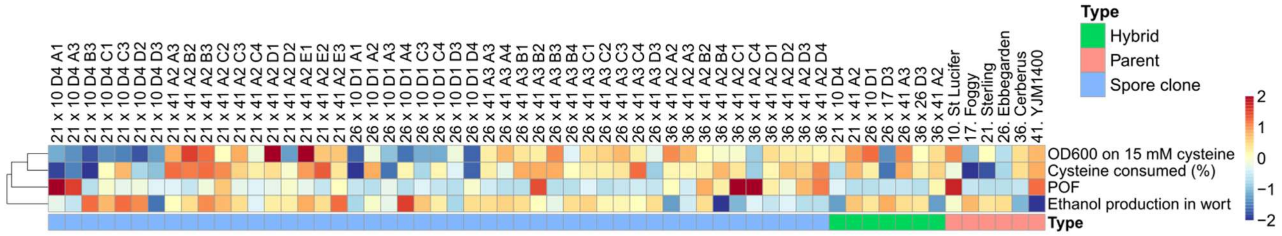

Supplementary Figure S4 - Phenotypic variation among the parent, hybrid, and spore clone strains. The heatmap is colored based on Z-scores (blue to red).

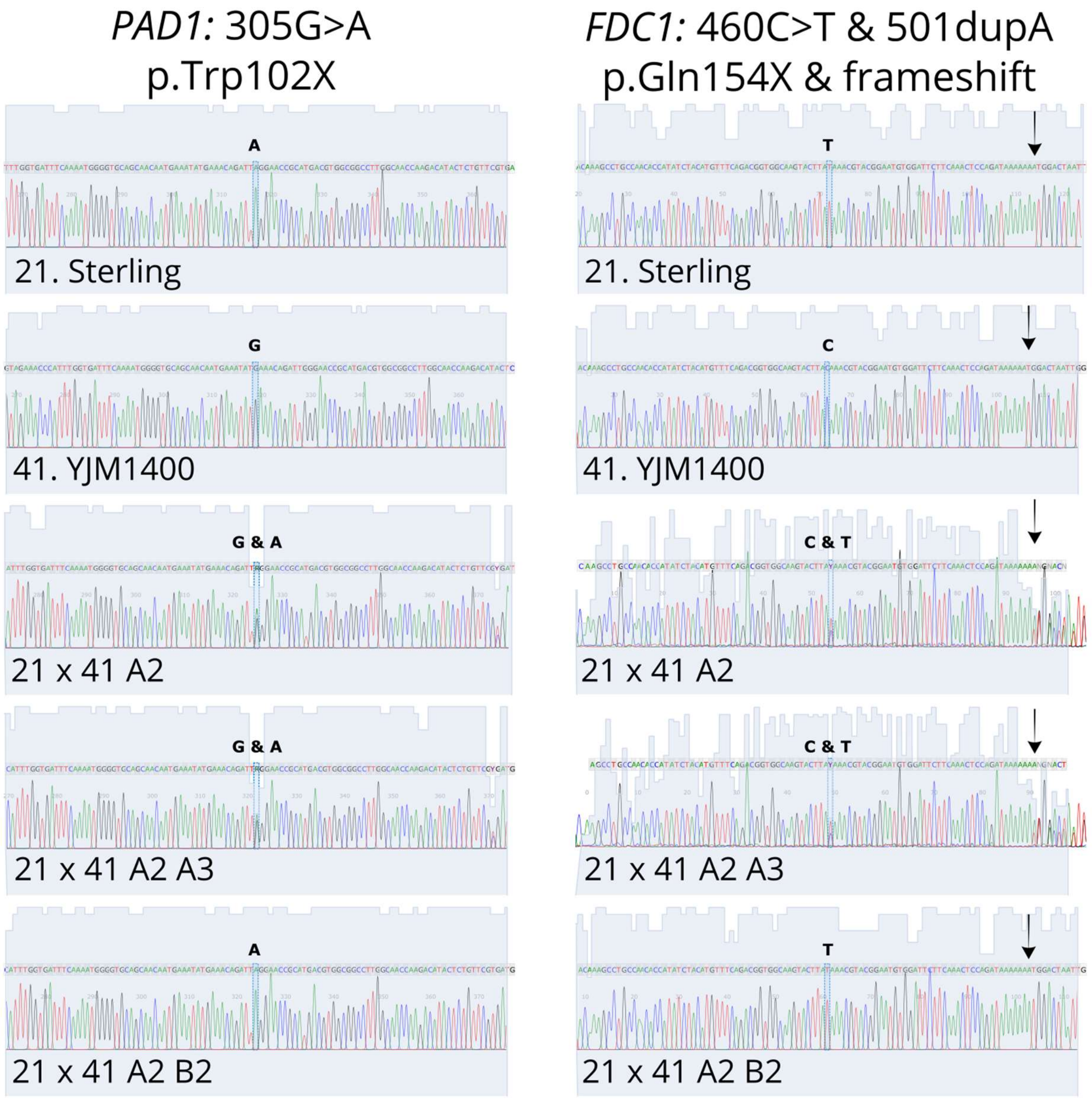

Supplementary Figure S5 - Inactivating mutations in *PAD1* and *FDC1* among parent, hybrid, and selected spore clone strains. Strains 21 × 41 A2 and 21 × 41 A2 A3 are heterozygous for the inactivating mutations, while 21 × 41 A2 B2 is homozygous. The heterozygous frameshift mutation in *FDC1* can be seen by the stretch of overlapping peaks.

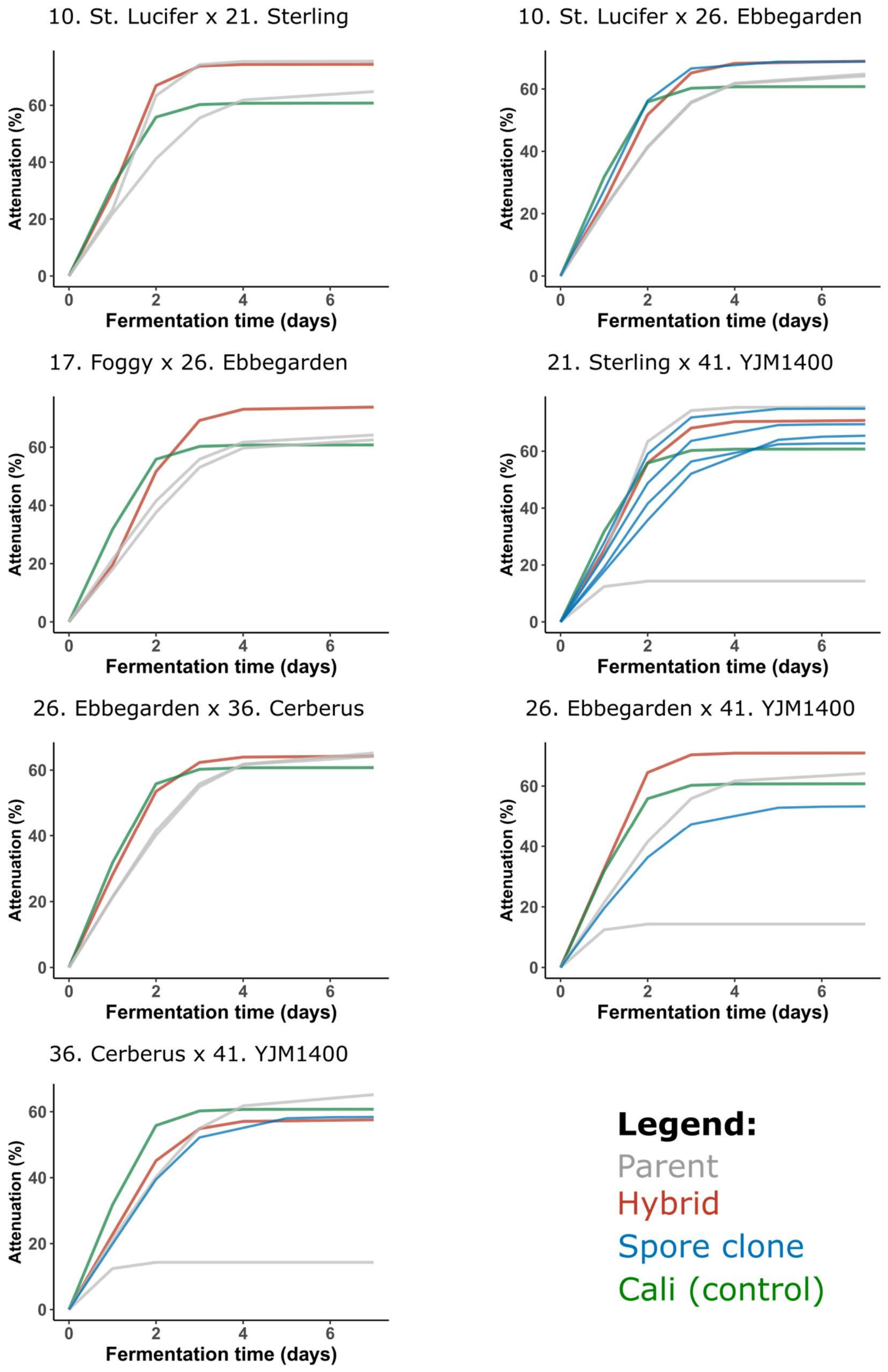

Supplementary Figure S6 - Screening of parent, hybrid and spore clone strains in 400 mL wort fermentations. Curves depict the apparent attenuation (%) during fermentations. Curves are colored according to strain type (grey: parent strains, red: F1 hybrids, and blue: spore clones, green: Cali Ale control).
